# Comparative Study of Postural Garment Versus Exercises for Patients With Nonspecific Cervical Pain: Protocol for a Randomized Crossover Trial

**DOI:** 10.2196/14807

**Published:** 2020-04-16

**Authors:** Merce Avellanet, Anna Boada-Pladellorens, Jean-Claude Perrot, Laura Loro, Lidia Rodrigo Cansado, David Monterde, Josep Romagosa, Elvira Gea

**Affiliations:** 1 Rehabilitation Department Hospital Nostra Senyora de Meritxell Escaldes- Engordany Andorra; 2 Department of Health Catalan Health Institute Govern de Catalunya Barcelona Spain; 3 Statistics Department Govern d'Andorra Andorra la Vella Andorra; 4 Pharmacy Department Hospital Nostra Senyora de Meritxell Escaldes- Engordany Andorra

**Keywords:** cervical pain, postural garment, cervical exercises, posture, musculoskeletal disorder

## Abstract

**Background:**

There is a high prevalence of work-related musculoskeletal disorders among health care professionals. Posture is an essential point to be addressed for health care professionals with musculoskeletal disorders. Cervical pain can result from several conditions. Treatment should include posture modification and home exercise.

**Objective:**

This study aims to compare a new postural garment (Posture Plus Force; Medi, Bayreuth, Germany) with exercises for women with nonspecific cervical pain. The investigators focus on nurses and allied health professionals due to the importance of posture in work-related musculoskeletal disorders.

**Methods:**

This randomized crossover clinical trial has a 3-month treatment sequence and a 3-month washout period. Participants will include nurses and allied health professionals 21 to 55 years of age with cervical pain. Participants are allocated at random to two intervention groups: a postural garment (Posture Plus Force) to be worn for 2 to 4 hours per day for 90 days (P+ group) and five physiotherapy sessions (20 minutes each) to learn stretching and strengthening exercises with instructions to continue at home on a daily basis for 90 days (Ex group). The participants in each group will crossover interventions after a 3-month washout period. The primary outcomes are postural control and pain intensity. A static posturography will be performed with a scan (SpinalMouse; Idiag AG, Fehraltorf, Switzerland). The visual analogue scale is a psychometric measuring instrument designed to document cervical pain severity in individual participants. The secondary outcomes are cervical pain-related disability, catastrophizing, the global perceived effect of treatment, and the evaluation of garment comfort. Physical activity is assessed with the International Physical Activity Questionnaire. Assessment of primary and secondary outcomes is performed at T0 (pre-intervention), T1 (immediately after garment fitting for P+ group), T30, T60, and T90. The same measurements are recorded after the washout period and during the second intervention following the same sequence. All patients are provided with a logbook for compliance recording, over the counter drug use, pain evaluation, and sick leave. Statistical analysis is conducted following intention-to-treat principles and the treatment effects calculated using linear mixed models.

**Results:**

The study design has been approved by the Ethics Commission of Hospital N Sra de Meritxell, Andorra in March 2017. A total of 32 participants are already enrolled in the study. An extension of the study is planned in a Spanish university hospital to achieve a larger sample. Study results are expected to be published during 2020.

**Conclusions:**

The Postural garment is expected to improve cervical pain by enhancing posture.

**Trial Registration:**

ClinicalTrials.gov NCT03560492; https://clinicaltrials.gov/ct2/show/NCT03560492

**International Registered Report Identifier (IRRID):**

DERR1-10.2196/14807

## Introduction

### Cervical Pain

There is a high prevalence of work-related musculoskeletal disorders among health care professionals. For instance, dental practitioners have reported a high prevalence of pain in the neck and shoulder region with rates in 2009 as high as 83% [1]. In our environment, and more concretely in our hospital, we found that during a 1-year period, 18% of the hospital staff were treated in the rehabilitation department, with more than 50% of cases caused by pain in the neck and back [2].

According to the Occupational Safety and Health Administration, musculoskeletal disorders affect the muscles, nerves, blood vessels, ligaments, and tendons [3]. Workers can be exposed to risk factors at work, such as lifting heavy items, bending, reaching overhead, pushing and pulling heavy loads, working in awkward body postures, and performing the same or similar tasks repetitively. In general, the more extreme the posture, the greater force required to complete the task.

In a recent study on dentists, a kinematic analysis underlined the posture in the area of the cervical and thoracic spine [4]. In addition, Freitag et al [5] demonstrated that sagittal trunk inclinations are an important factor in the evaluation of physical strain in nursing staff. All in all, posture is an essential point to be addressed for musculoskeletal disorders in health care professionals.

Cervical pain can result from several conditions. Disorders that cause axial neck pain include cervical strain, internal disc disruption syndrome, cervical facet-mediated pain, cervical “whiplash” syndrome, and myofascial pain. Treatment for neck pain is distinct from treatment for extremity pain or symptoms. Most patients with mild to moderate axial pain improve in 2 to 3 weeks. Treatment should include posture modification and home exercise [6-8].

### Postural Garment

The Posture Plus System adopts advanced solutions focusing on the abdomen, shoulders, and dorsal areas. The Posture Plus Force (Medi, Bayreuth, Germany) is based on the lessons learned from the development of the spinal orthosis Spinomed (Medi, Bayreuth, Germany) regarding strength and posture [9]. The Posture Plus Force is a T-shirt that includes tensional inelastic bands exercising traction for postural realignment. There are different garments available with this aim in the market. However, the Posture Plus Force is unique due to its design. Enhancement of posture is centered using an anatomical and physiological approach that focuses on abdominal and periscapular muscles.

**Figure 1:**
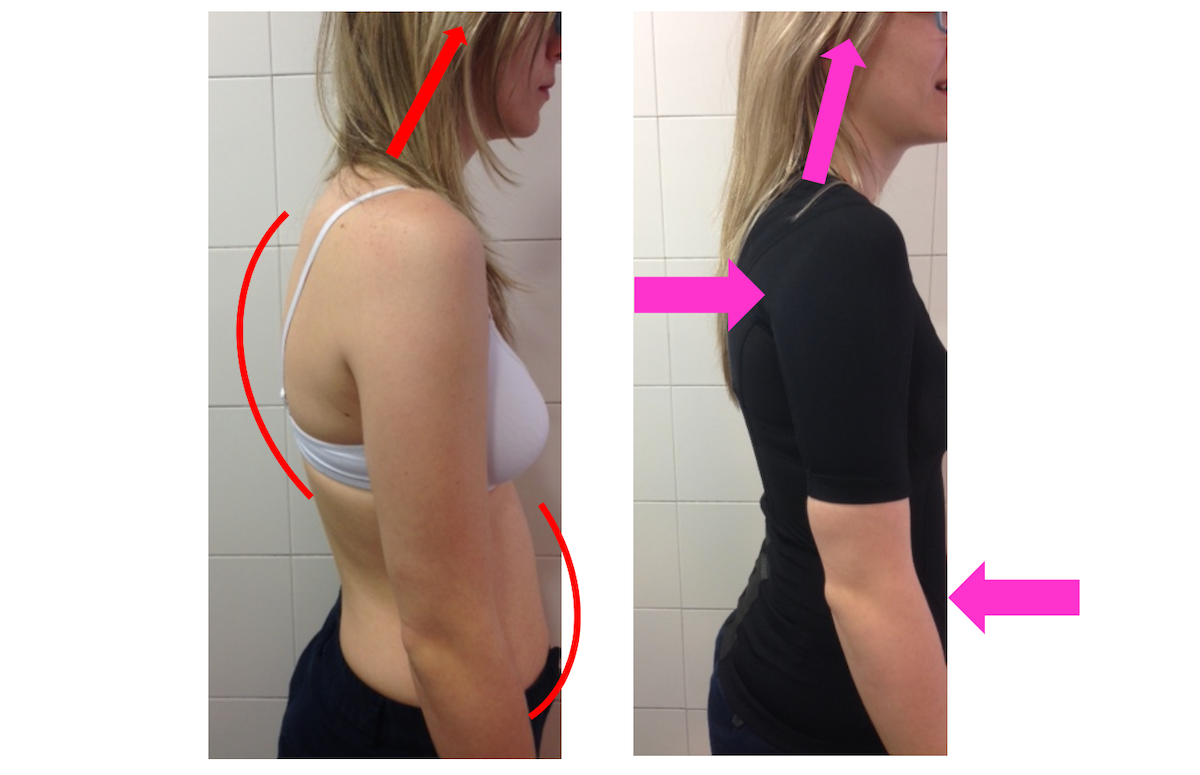
Posture Plus Force garment.

We have tested the garment in a few patients with dorsal hyperkyphosis. We have noticed that participants reported improvements in dorsal symptoms and cervical well-being. The abdominal inelastic bands enhance core muscle contractions that facilitate a correct upright position and realign the cervical segment as well. Moreover, the dorsal bands are positioned in a functional anatomical direction following interscapular forces and enhancing perivertebral contractions instead of pulling back shoulders as other garments tend to do. Both tensional bands improve spine alignment from the abdominal zone to the cephalic position, including dorsal and cervical areas (see Figure 1). People, who do not usually remain consistent in their exercises, improved their posture and maintained a better posture during and after wearing the postural garment.

Given the high prevalence of cervical pain in health professionals previously mentioned, we hypothesized that the use of this garment can be useful for this pathology.

### Hypothesis

The postural garment will improve cervical pain by enhancing posture.

## Methods

Inclusion and exclusion criteria for our study include the following.

### Inclusion Criteria

- Nurses and health allied professionals with cervical pain ≥3 on the visual analogue scale (VAS)
- Women 21 to 55 years of age
- Voluntarily signed informed consent
- Able to wear the garment and perform exercises, and attend follow-up assessments

### Exclusion Criteria

- Pregnancy
- Malignancy or other severe disease
- Cervical pain with significant extremity symptoms or neurological dysfunctions (cervical radiculopathy and cervical spondylotic myelopathy)
- Unable to perform exercises
- Unwilling to do follow-up assessments
- Psychiatric disorders

Participants will be allocated at random to receive interventions in two groups based on a randomization plan.

One group will wear the postural garment (P+ group). The postural garment (Posture Plus Force) is provided to participants. It has to be worn for 2 to 4 hours per day for 90 days. See Figure 1.

The exercise group (Ex group) will do 5 physiotherapy sessions (20 minutes each) to learn stretching and strengthening exercises for the cervical and dorsal areas, with instructions to continue at home on a daily basis for 90 days. The precise explanation of exercises is shown in Multimedia Appendix 1. Home exercise maintains range of motion and helps patients become active participants in their care.

After a 3-month washout period to reverse any change that may have occurred with the first intervention, the participants will crossover the intervention for another 3-month period.

### Collected Variables

The following factors will be assessed for all participants: age, weight, height, BMI, work experience, part-time employment, education, managerial position, pharmacological treatment, global perceived effect of treatment, treatment compliance, and garment comfort.

The level of physical activity is evaluated with the International Physical Activity Questionnaire (IPAQ).

Education will be divided into registered nurse, nonregistered nurse, and allied health professional. Managerial positions will include ward managers and group managers. Pharmacological treatment and sick leave will be recorded when necessary. Other variables will include main and secondary outcome measures.

### Outcome Measures

The primary outcomes will be postural control and pain intensity.

Posture can be evaluated with pictures on the sagittal and frontal plane. Objective measurements of static posturography will be performed with a scan (SpinalMouse; Idiag AG, Fehraltorf, Switzerland). SpinalMouse is a device that, when combined with a PC program, assesses the curvatures of the spine without applying radiation. The device is guided manually on the skin along the spinous process apophysis from C7 to S3. The measuring head follows the contour in the sagittal plane and records clinically relevant data. A software program using a highly sophisticated algorithm uses this information to calculate the clinical parameters. The data obtained have been proven reliable and accurate [10,11] in different population groups [12-14]. The standardization and validated protocol do not include the cervical region, but dorsal kyphosis and lumbar lordosis angles influence the cervical position.

To blind the assessment of posture, the investigator that performs the SpinalMouse scan will not be aware of the participants’ current intervention at each follow-up.

All other outcomes will be recorded by the other investigators.

Pain will be measured by the VAS. The VAS is a psychometric measuring instrument designed to document cervical pain severity in individual participants. It achieves a rapid (statistically measurable and reproducible) classification of symptom severity. A 100 mm horizontal line with verbal descriptors will be used to grade the amount of pain that a patient feels from no pain (left 0 mm) to an extreme amount of pain (right 100 mm).

Secondary outcomes will include cervical pain-related disability, catastrophizing, the global perceived effect of treatment, and the evaluation of garment comfort for the P+ group. Days out of work will be recorded in cases of sick leave.

Cervical pain-related disability will be assessed with the validated Spanish version of the Neck Disability Index (Multimedia Appendix 2). The scale includes 10 questions on different aspects of disability due to cervical pain [15].

Psychosocial factors are significant in predicting the duration and severity of symptoms. A significant relation between catastrophizing and pain-related outcomes has been observed in numerous pain samples [16]. Sullivan et al [17] defined catastrophizing as “an exaggerated negative mental set brought to bear during actual or anticipated painful experience”. Individuals who score high on measures of pain catastrophizing report more intense pain. To assess this dimension related to pain, we will use the Pain Catastrophizing Scale (PCS) (Multimedia Appendix 3). The PCS is currently one of the most widely used measures of catastrophic thinking related to pain. It is a 13-item instrument that yields a total score and three subscale scores assessing rumination, magnification, and helplessness.

Assessment of the global perceived effect of treatment will be performed with a VAS (Multimedia Appendix 4). Garment comfort will be evaluated with a 5-point Likert scale question, an open question, and free comments (Multimedia Appendix 5).

Physical activity will be assessed with a self-reported physical activity questionnaire, the Spanish version of the IPAQ short form [18,19].

The assessment of primary and secondary outcomes will be performed at T1, T30, T60, and T90. The same measurements will be recorded after the washout period during the crossover period treatment (T0b, T30b, T60b, T90b). As previously mentioned, the same investigators will follow up with participants except for the SpinalMouse measurement, which will be performed by a blind collaborator.

All patients will be provided with a logbook for compliance recording, over the counter drug use, pain evaluation, and sick leave.

### Statistical Analysis

Statistical analyses will be conducted following intention-to-treat principles, and the treatment effects will be calculated using linear mixed models. Sample size is determined with a 2 point expected difference in the VAS for pain and a sample standard deviation of 2.5.

## Results

The study design has been approved by the Ethics Commission of Hospital N Sra de Meritxell, Andorra in March 2017. A total of 32 participants are already enrolled in the study. An extension of the study is planned in a Spanish university hospital to obtain a larger sample. Study results are expected to be published during 2020.

## Discussion

### Cervical Pain and Posture

The main component of cervical pain management is exercise. A correct posture is maintained with core muscle contractions. The Posture Plus Force postural garment may enhance posture by muscle activation. Both exercise and postural improvement are expected to improve cervical pain.

### Potential Risks

No significant risks can emerge from this protocol study.

All patients will be assessed before being included and during the follow-up by a physical medicine and rehabilitation physician. Exercises will be taught by a certified physiotherapist.

Regarding the suitability of interventions, it has been stated that usual physiotherapy may be only marginally better than brief physiotherapy intervention (1-3 sessions) for neck pain [20]. According to our clinical practice, any patient will be free to schedule an earlier appointment if warranted during the study period.

### Ethical Issues

The study design has been approved by the Ethics Commission of Hospital N Sra de Meritxell, Andorra. The standards of the CONSORT (Consolidated Standards of Reporting Trials) statement of 1996, as revised in 2010, will be followed.

Prior to being enrolled in the study, all patients will be informed about the study objectives, measurements and pictures to be taken. All participants have to give their consent to being photographed in advance. All the participants have to sign the informed consent before enrollment.

### Financial Disclosure

No funding is received by investigators or participants.

Medi (Bayreuth, Germany) provides the logbook and postural garments for all participants.

This study has the approval of the hospital medical director. Costs will be recorded and calculated for both interventions. In our environment, typical protocol treatment for cervical pain includes 2 to 3 consultations with a physical medicine and rehabilitation physician, and 15 to 20 physiotherapy sessions. For this study protocol, assessment visits will be higher than the standard procedure, but physiotherapy sessions will dramatically diminish. Moreover, participants will aim to be an active part of their neck pain care. In conclusion, we expect a decrease in costs compared to usual cervical pain approaches in our department combined with the educational self-management of pain.

## Appendix

Multimedia Appendix 1 Exercise protocol.

Multimedia Appendix 2 Spanish version of Neck Disability Index.

Multimedia Appendix 3 Pain Catastrophizing Scale Spanish version.

Multimedia Appendix 4 Assessment of global perceived effect of treatment.

Multimedia Appendix 5 Garment comfort evaluation.

## Abbreviations

CONSORT: Consolidated Standards of Reporting Trials
IPAQ: International Physical Activity Questionnaire
PCS: Pain Catastrophizing Scale
VAS: visual analogue scale.

## References

[1] Morse T, Bruneau H, Dussetschleger J (2010). Musculoskeletal disorders of the neck and shoulder in the dental professions. Work.

[2] Avellanet Viladomat M, Sáenz Guerrero A, Hijós Bitrián E, Romagosa Massana J (2005). Asistencia al personal hospitalario en un servicio de rehabilitación. ¿Cómo es y cuánto cuesta?. Rehabilitación.

[3] Occupational Safety and Health Administration OSHA.gov.

[4] Ohlendorf D, Erbe C, Hauck I, Nowak J, Hermanns I, Ditchen D, Ellegast R, Groneberg DA (2016). Kinematic analysis of work-related musculoskeletal loading of trunk among dentists in Germany. BMC Musculoskelet Disord.

[5] Freitag S, Fincke-Junod I, Seddouki R, Dulon M, Hermanns I, Kersten JF, Larsson TJ, Nienhaus A (2012). Frequent bending--an underestimated burden in nursing professions. Ann Occup Hyg.

[6] Isaac Z, Atlas SJ, Dashe J (2016). Treatment of neck pain. UpToDate.

[7] Gross A, Kay TM, Paquin J, Blanchette S, Lalonde P, Christie T, Dupont G, Graham N, Burnie SJ, Gelley G, Goldsmith CH, Forget M, Hoving JL, Brønfort G, Santaguida PL, Cervical Overview Group (2015). Exercises for mechanical neck disorders. Cochrane Database Syst Rev.

[8] Im B, Kim Y, Chung Y, Hwang S (2016). Effects of scapular stabilization exercise on neck posture and muscle activation in individuals with neck pain and forward head posture. J Phys Ther Sci.

[9] Pfeifer M, Kohlwey L, Begerow B, Minne HW (2011). Effects of two newly developed spinal orthoses on trunk muscle strength, posture, and quality-of-life in women with postmenopausal osteoporosis: a randomized trial. Am J Phys Med Rehabil.

[10] Post RB, Leferink VJM (2004). Spinal mobility: sagittal range of motion measured with the SpinalMouse, a new non-invasive device. Arch Orthop Trauma Surg.

[11] Mannion AF, Knecht K, Balaban G, Dvorak J, Grob D (2004). A new skin-surface device for measuring the curvature and global and segmental ranges of motion of the spine: reliability of measurements and comparison with data reviewed from the literature. Eur Spine J.

[12] Lang-Tapia M, España-Romero Vanesa, Anelo J, Castillo MJ (2011). Differences on spinal curvature in standing position by gender, age and weight status using a noninvasive method. J Appl Biomech.

[13] Mizukami S, Abe Y, Tsujimoto R, Arima K, Kanagae M, Chiba G, Aoyagi K (2014). Accuracy of spinal curvature assessed by a computer-assisted device and anthropometric indicators in discriminating vertebral fractures among individuals with back pain. Osteoporos Int.

[14] Livanelioglu A, Kaya F, Nabiyev V, Demirkiran G, Fırat T (2016). The validity and reliability of "Spinal Mouse" assessment of spinal curvatures in the frontal plane in pediatric adolescent idiopathic thoraco-lumbar curves. Eur Spine J.

[15] Ortega JA, Martínez AD, Ruiz R (2010). Validation of the Spanish version of the Neck Disability Index. Spine (Phila Pa 1976).

[16] Nordin CA, Michaelson P, Gard G, Eriksson MK (2016). Effects of the web behavior change program for activity and multimodal pain rehabilitation: randomized controlled trial. J Med Internet Res.

[17] Sullivan MJ, Thorn B, Haythornthwaite JA, Keefe F, Martin M, Bradley LA, Lefebvre JC (2001). Theoretical perspectives on the relation between catastrophizing and pain. Clin J Pain.

[18] Roman-Viñas B, Serra-Majem L, Hagströmer M, Ribas-Barba L, Sjöström M, Segura-Cardona R (2010). International Physical Activity Questionnaire: reliability and validity in a Spanish population. European Journal of Sport Science.

[19] Silsbury Z, Goldsmith R, Rushton A (2015). Systematic review of the measurement properties of self-report physical activity questionnaires in healthy adult populations. BMJ Open.

[20] Klaber Moffett JA, Jackson DA, Richmond S, Hahn S, Coulton S, Farrin A, Manca A, Torgerson DJ (2005). Randomised trial of a brief physiotherapy intervention compared with usual physiotherapy for neck pain patients: outcomes and patients' preference. BMJ.

