# Supplementary material for: Comparative Study of Postural Garment Versus Exercises for Patients With Nonspecific Cervical Pain: Protocol for a Randomized Crossover Trial
Source: JMIR Res Protoc. 2020 Apr 16;9(4):e14807. doi: 10.2196/14807 (PMC7193442; doi:10.2196/14807)
Supplement: Multimedia Appendix 1 [file resprot_v9i4e14807_app1.pptx]

## Slide 1
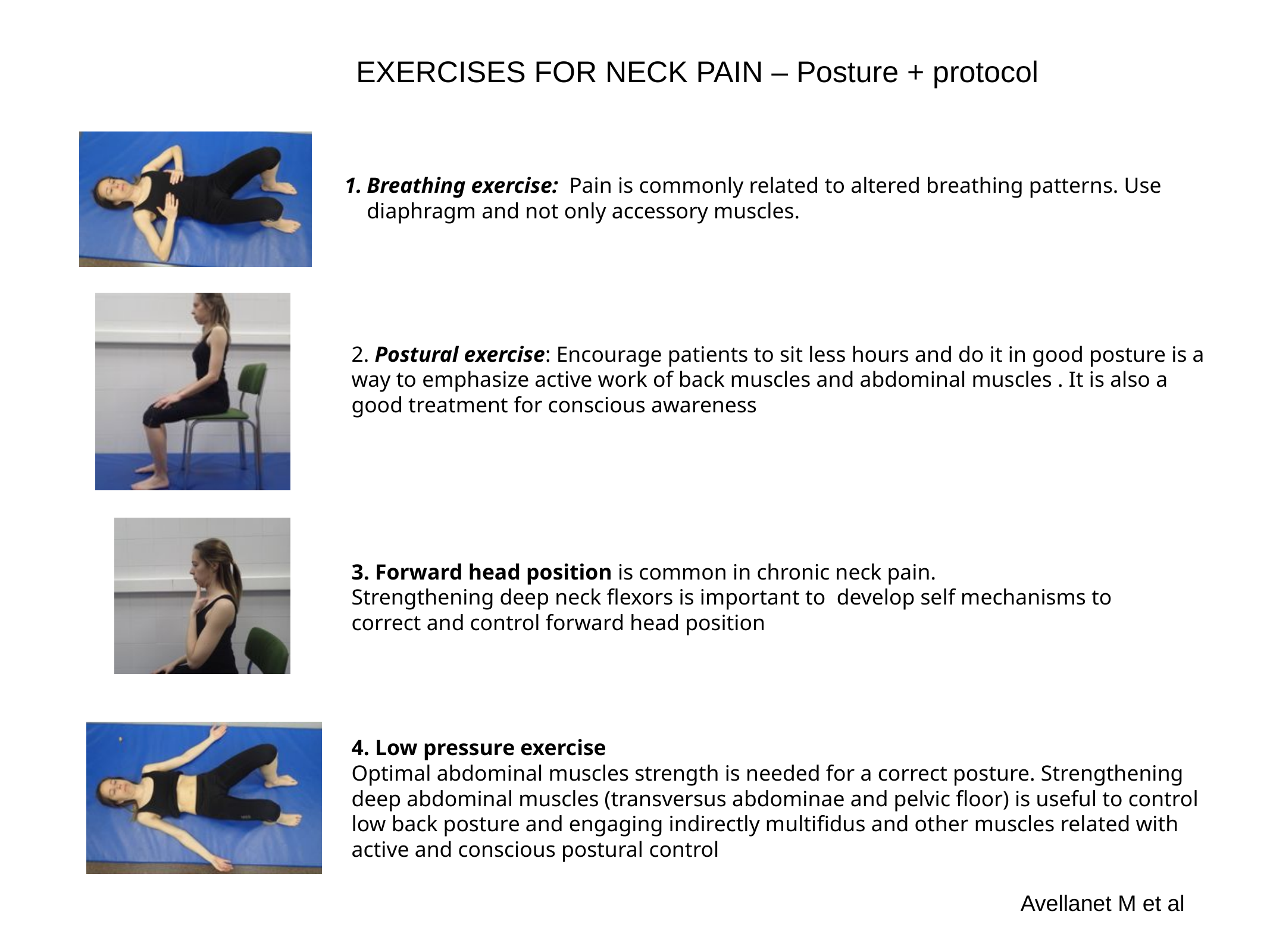

EXERCISES FOR NECK PAIN – Posture + protocol
Breathing exercise: Pain is commonly related to altered breathing patterns. Use diaphragm and not only accessory muscles.
2. Postural exercise: Encourage patients to sit less hours and do it in good posture is a way to emphasize active work of back muscles and abdominal muscles . It is also a good treatment for conscious awareness
3. Forward head position is common in chronic neck pain.
Strengthening deep neck flexors is important to develop self mechanisms to correct and control forward head position
4. Low pressure exercise
Optimal abdominal muscles strength is needed for a correct posture. Strengthening deep abdominal muscles (transversus abdominae and pelvic floor) is useful to control low back posture and engaging indirectly multifidus and other muscles related with active and conscious postural control
Avellanet M et al

## Slide 2
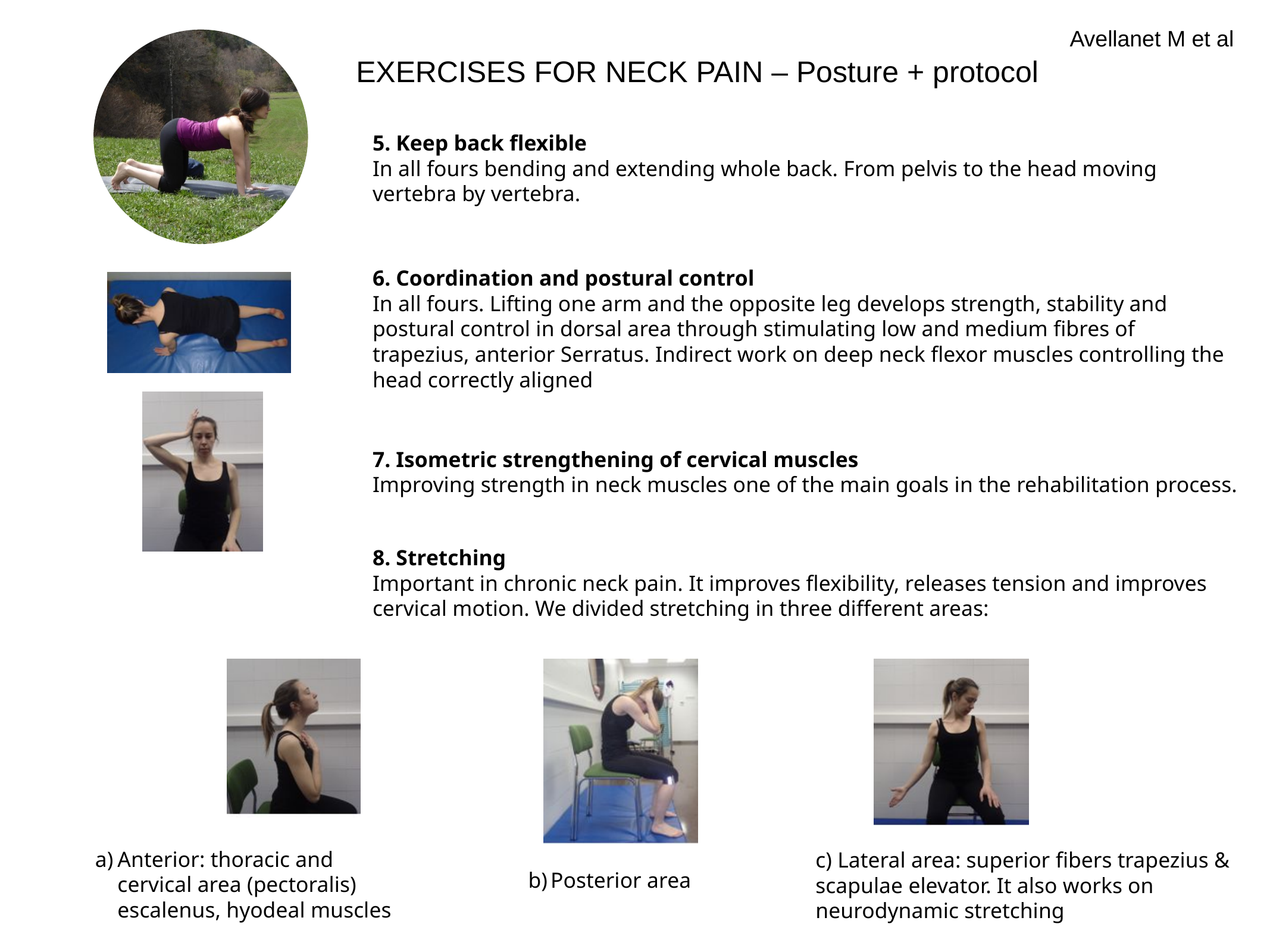

Avellanet M et al
EXERCISES FOR NECK PAIN – Posture + protocol
5. Keep back flexible
In all fours bending and extending whole back. From pelvis to the head moving vertebra by vertebra.
6. Coordination and postural control
In all fours. Lifting one arm and the opposite leg develops strength, stability and postural control in dorsal area through stimulating low and medium fibres of trapezius, anterior Serratus. Indirect work on deep neck flexor muscles controlling the head correctly aligned
7. Isometric strengthening of cervical muscles
Improving strength in neck muscles one of the main goals in the rehabilitation process.
8. Stretching
Important in chronic neck pain. It improves flexibility, releases tension and improves cervical motion. We divided stretching in three different areas:
Anterior: thoracic and cervical area (pectoralis) escalenus, hyodeal muscles
c) Lateral area: superior fibers trapezius & scapulae elevator. It also works on neurodynamic stretching
Posterior area
