# Supplementary material for: Comparative Study of Postural Garment Versus Exercises for Patients With Nonspecific Cervical Pain: Protocol for a Randomized Crossover Trial
Source: JMIR Res Protoc. 2020 Apr 16;9(4):e14807. doi: 10.2196/14807 (PMC7193442; doi:10.2196/14807)
Supplement: Multimedia Appendix 2 [file resprot_v9i4e14807_app2.docx]

### Appendix 2. Spanish version of Neck Disability Index.

Scoring and interpretation.

**NECK DISABILITY INDEX QUESTIONNAIRE**

Patient Name _________________________________________ Date ___________

Examiner ____________________________________________

Este cuestionario se ha diseñado para permitirnos entender como le afecta a su vida diaria el dolor de cuello. Por favor responda todas las preguntas posibles y marque en cada una **SOLO UNA RESPUESTA** la que más se aproxime a su caso.

1. Intensidad del dolor de cuello

No tengo dolor en este momento.

El dolor es muy leve en este momento.

El dolor es moderado en este momento.

El dolor es fuerte en este momento.

El dolor es muy fuerte en este momento.

En este momento el dolor es el peor que uno se puede imaginar.

2. Cuidados personales (lavarse, vestirse, etc.)

Puedo cuidarme con normalidad sin que me aumente el dolor.

Puedo cuidarme con normalidad, pero esto me aumenta el dolor.

Cuidarme me duele de forma que tengo que hacerlo despacio y con cuidado.

Aunque necesito alguna ayuda, me las arreglo para casi todos mis cuidados.

Todos los días necesito ayuda para la mayor parte de mis cuidados.

No puedo vestirme, me lavo con dificultad y me quedo en la cama.

3. Levantar pesos

Puedo levantar objetos pesados sin aumento del dolor.

Puedo levantar objetos pesados, pero me aumenta el dolor.

El dolor me impide levantar objetos pesados del suelo, pero lo puedo hacer si están colocados en un sitio fácil como, por ejemplo, en una mesa.

El dolor me impide levantar objetos pesados del suelo, pero puedo levantar objetos medianos o ligeros si están colocados en un sitio fácil.

Sólo puedo levantar pesos muy ligeros

No puedo levantar ni llevar ningún tipo de peso.

4. Lectura

Puedo leer todo lo que quiera sin que me duela el cuello

Puedo leer todo lo que quiera con un dolor leve en el cuello

Puedo leer todo lo que quiera con un dolor moderado en el cuello

No puedo leer todo lo que quiero debido a un dolor moderado en el cuello

Apenas puedo leer por el gran dolor que me produce en el cuello

No puedo leer nada en absoluto

5. Dolor de cabeza

No tengo ningún dolor de cabeza.

A veces tengo un pequeño dolor de cabeza.

A veces tengo un dolor moderado de cabeza.

Con frecuencia tengo un dolor moderado de cabeza.

Con frecuencia tengo un dolor fuerte de cabeza.

Tengo dolor de cabeza casi continuo.

6. Concentrarse en algo

Me concentro totalmente en algo cuando quiero sin dificultad.

Me concentro totalmente en algo cuando quiero con alguna dificultad.

Tengo alguna dificultad para concentrarme cuando quiero.

Tengo bastante dificultad para concentrarme cuando quiero.

Tengo mucha dificultad para concentrarme cuando quiero.

No puedo concentrarme nunca.

7. Trabajo y actividades habituales

Puedo trabajar todo lo que quiero.

Puedo hacer mi trabajo habitual, pero no más.

Puedo hacer casi todo mi trabajo habitual, pero no más.

No puedo hacer mi trabajo habitual.

A duras penas puedo hacer algún tipo de trabajo.

No puedo trabajar en nada.

8. Conducción de vehículos

Puedo conducir sin dolor de cuello.

Puedo conducir todo lo que quiero, pero con un ligero dolor de cuello.

Puedo conducir todo lo que quiero, pero con un moderado dolor de cuello.

No puedo conducir todo lo que quiero debido al dolor de cuello.

Apenas puedo conducir debido al intenso dolor de cuello.

No puedo conducir nada por el dolor de cuello.

9. Sueño

No tengo problemas para dormir

El dolor de cuello me hace perder menos de 1 hora de sueño cada noche.

El dolor de cuello me hace perder de 1 a 2 horas de sueño cada noche.

El dolor de cuello me hace perder de 2 a 3 horas de sueño cada noche.

El dolor de cuello me hace perder de 3 a 5 horas de sueño cada noche.

El dolor de cuello me hace perder de 5 a 7 horas de sueño cada noche.

10. Actividades de ocio

Puedo hacer todas mis actividades de ocio sin dolor de cuello

Puedo hacer todas mis actividades de ocio con algún dolor de cuello.

No puedo hacer algunas de mis actividades de ocio por el dolor de cuello

Sólo puedo hacer unas pocas actividades de ocio por el dolor del cuello.

Apenas puedo hacer las cosas que me gustan debido al dolor del cuello.

No puedo realizar ninguna actividad de ocio.

**SCORING AND INTERPRETATION**

The Neck Disability Index (NDI) was developed in 1989 by Howard Vernon. The Index was developed as a modification of the Oswestry Low Back Pain Disability Index with the permission of the original author (J. Fairbank, 1980). In 1991, Vernon and Mior published the results of a study of reliability and validity in the Journal of Manipulative and Physiologic Therapeutics. Since then, approximately ten articles have appeared in the indexed literature on the NDI. All of these studies have confirmed the original reports of a high level of reliability and validity. We currently know that the NDI consists of one factor - “physical disability” - although NDI scores correlate well with SF-36 mental component scores as well. We know that the minimum detectable score and the minimal clinically important difference amount to the same figure - 5 NDI points. The NDI has become a standard instrument for measuring self-rated disability due to neck pain and is used by clinicians and researchers alike.

**Scoring**

Each of the 10 items is scored from 0 - 5. The maximum score is therefore 50. The obtained score can be multiplied by 2 to produce a percentage score. Occasionally, a respondent will not complete one question or another. The average of all other items is then added to the completed items.

**The original report provided scoring intervals for interpretation, as follows:**

**0 - 4 = no disability**

**5 - 14 = mild**

**15 - 24 = moderate**

**25 - 34 = severe**

**Above 34 = complete.**

**Please note:**

**This means 15-24 out of 50 (the RAW SCORE) equates with moderate disability.**

It is recommended that the NDI be used at baseline and for every 2 weeks thereafter within the treatment program to measure progress. As noted above, at least a 5-point change is required to be clinically meaningful. Patients often do not score the items as zero, once they are in treatment. In other words, it is common to find that patients will continue to score between 5 - 15 despite having made excellent recovery (i.e., they may be back to work). The practitioner should avoid the trap of “treating till zero”, as this is not supportable based on current evidence.

**Interpretation of Neck Disability Index Scores**

**Raw Score Relative Impairment**

0-4 None

5-14 Mild

15-24 Moderate

25-34 Severe

> 35 Complete

Source: Vernon, H. and Mior, S. The Neck Disability Index: A study of reliability and validity. *Journal of Manipulative and Physiological Therapeutics*, 1991, 14, 409-415.
