# Supplementary material for: Comparative Study of Postural Garment Versus Exercises for Patients With Nonspecific Cervical Pain: Protocol for a Randomized Crossover Trial
Source: JMIR Res Protoc. 2020 Apr 16;9(4):e14807. doi: 10.2196/14807 (PMC7193442; doi:10.2196/14807)
Supplement: Multimedia Appendix 3 [file resprot_v9i4e14807_app3.docx]

### Appendix 3. Pain Catastrophizing Scale Spanish version.

Todas las personas experimentamos situaciones de dolor en algún momento de nuestra vida. Tales experiencias pueden incluir dolor de cabeza, dolor de muelas, dolor muscular o de articulaciones. Las personas estamos a menudo expuestas a situaciones que pueden causar dolor como las enfermedades, las heridas, los tratamientos dentales o las intervenciones quirúrgicas. Estamos interesados en conocer el tipo de pensamientos y sentimientos que usted tiene cuando siente dolor. A continuación se presenta una lista de 13 frases que describen diferentes pensamientos y sentimientos que pueden estar asociados al dolor. Utilizando la siguiente escala, por favor, indique el grado en que usted tiene esos pensamientos y sentimientos cuando siente dolor.

0: Nada en absoluto 1: Un poco 2: Moderadamente 3: Mucho 4: Todo el tiempo

| Preguntas | Valor |
| --- | --- |
| 1. Estoy preocupado todo el tiempo pensando en si el dolor desaparecerá |  |
| 1. Siento que ya no puedo más |  |
| 3. Es terrible y pienso que esto nunca va a mejorar |  |
| 4. Es horrible y siento que esto es más fuerte que yo |  |
| 5. Siento que no puedo soportarlo más |  |
| 6. Temo que el dolor empeore |  |
| 7. No dejo de pensar en otras situaciones en las que experimento dolor |  |
| 8. Deseo desesperadamente que desaparezca el dolor |  |
| 9. No puedo apartar el dolor de mi mente |  |
| 10. No dejo de pensar en lo mucho que me duele |  |
| 11. No dejo de pensar en lo mucho que deseo que desaparezca el dolor |  |
| 12. No hay nada que pueda hacer para aliviar la intensidad del dolor |  |
| 13. Me pregunto si me puede pasar algo grave |  |
| TOTAL |  |
