# Supplementary material for: Comparative Study of Postural Garment Versus Exercises for Patients With Nonspecific Cervical Pain: Protocol for a Randomized Crossover Trial
Source: JMIR Res Protoc. 2020 Apr 16;9(4):e14807. doi: 10.2196/14807 (PMC7193442; doi:10.2196/14807)
Supplement: Multimedia Appendix 4 [file resprot_v9i4e14807_app4.docx]

### Appendix 4. Assessment of global perceived effect of treatment.

Estudi ***Posture: Estudi comparatiu de una prenda postural versus exercicis per pacients amb dolor cervical no específic.***

Valori si us plau l’efecte del tractament sobre el dolor cervical.

Marqui sobre la línea la milloria que ha notat.

Cap milloria Molta milloria

Ninguna mejoría Mucha mejoria

---------------------------------------------------------------------------------------
