# Supplementary material for: Comparative Study of Postural Garment Versus Exercises for Patients With Nonspecific Cervical Pain: Protocol for a Randomized Crossover Trial
Source: JMIR Res Protoc. 2020 Apr 16;9(4):e14807. doi: 10.2196/14807 (PMC7193442; doi:10.2196/14807)
Supplement: Multimedia Appendix 5 [file resprot_v9i4e14807_app5.docx]

### Appendix 5. Garment comfort evaluation.

Estudi ***Posture: Estudi comparatiu de una prenda postural versus exercicis per pacients amb dolor cervical no específic.***

*Volem saber si la prenda és confortable.*

*Valori si us plau, la confortabilitat de la camiseta que ha portat. Marqui el que s’ajusti més a la seva percepció.*

*molt incomoda incomoda indiferent còmoda molt còmoda*

*Ο Ο Ο Ο Ο*

*Si us plau, agrairem que ens faci suggeriments sobre la camiseta:*

*----------------------------------------------------------------------------------------------------------------------------------------------------------------------------------------------------------------------------------------------------------------------------------------------------------------------------------------------------------------------------------------------------------------------------------------*
